# Chemokine signals are crucial for enhanced homing and differentiation of circulating osteoclast progenitor cells

**DOI:** 10.1186/s13075-017-1337-6

**Published:** 2017-06-15

**Authors:** Alan Sucur, Zrinka Jajic, Marinko Artukovic, Marina Ikic Matijasevic, Branimir Anic, Darja Flegar, Antonio Markotic, Tomislav Kelava, Sanja Ivcevic, Natasa Kovacic, Vedran Katavic, Danka Grcevic

**Affiliations:** 1 Croatian Institute for Brain Research, University of Zagreb School of Medicine, Salata 12, Zagreb, HR 10000 Croatia; 2 Department of Physiology and Immunology, University of Zagreb School of Medicine, Salata 3b, Zagreb, HR 10000 Croatia; 3 Department of Rheumatology, Physical Medicine and Rehabilitation, Clinical Hospital Center “Sestre Milosrdnice”, University of Zagreb School of Medicine, Vinogradska cesta 29, Zagreb, HR 10000 Croatia; 4 Department of Clinical Immunology and Pulmonology, Clinical Hospital “Sveti Duh”, Sveti Duh 64, Zagreb, HR 10000 Croatia; 5 Department of Clinical Immunology and Rheumatology, Clinical Hospital Center “Zagreb”, Kispaticeva 12, Zagreb, HR 10000 Croatia; 6 Department of Anatomy, University of Zagreb School of Medicine, Salata 11, Zagreb, HR 10000 Croatia

**Keywords:** Osteoclast progenitor cells, Chemokines, Chemokine receptors, Osteoclasts, Rheumatoid arthritis, Peripheral blood, Synovial fluid, Cytokines, Inflammation, Bone loss

## Abstract

**Background:**

The peripheral blood (PB) monocyte pool contains osteoclast progenitors (OCPs), which contribute to osteoresorption in inflammatory arthritides and are influenced by the cytokine and chemokine milieu. We aimed to define the importance of chemokine signals for migration and activation of OCPs in rheumatoid arthritis (RA) and psoriatic arthritis (PsA).

**Methods:**

PB and, when applicable, synovial fluid (SF) samples were collected from 129 patients with RA, 53 patients with PsA, and 110 control patients in parallel to clinical parameters of disease activity, autoantibody levels, and applied therapy. Receptors for osteoclastogenic factors (CD115 and receptor activator of nuclear factor-κB [RANK]) and selected chemokines (CC chemokine receptor 1 [CCR1], CCR2, CCR4, CXC chemokine receptor 3 [CXCR3], CXCR4) were determined in an OCP-rich subpopulation (CD3^−^CD19^−^CD56^−^CD11b^+^CD14^+^) by flow cytometry. In parallel, levels of CC chemokine ligand 2 (CCL2), CCL3, CCL4, CCL5, CXC chemokine ligand 9 (CXCL9), CXCL10, and CXCL12 were measured using cytometric bead array or enzyme-linked immunosorbent assay. Sorted OCPs were stimulated in culture by macrophage colony-stimulating factor and receptor activator of nuclear factor-κB ligand, and they were differentiated into mature osteoclasts that resorb bone. Selected chemokines (CCL2, CCL5, CXCL10, and CXCL12) were tested for their osteoclastogenic and chemotactic effects on circulatory OCPs in vitro.

**Results:**

The OCP population was moderately enlarged among PB cells in RA and correlated with levels of tumor necrosis factor-α (TNF-α), rheumatoid factor, CCL2, and CCL5. Compared with PB, the RANK^+^ subpopulation was expanded in SF and correlated with the number of tender joints. Patients with PsA could be distinguished by increased RANK expression rather than total OCP population. OCPs from patients with arthritis had higher expression of CCR1, CCR2, CCR4, CXCR3, and CXCR4. In parallel, patients with RA had increased levels of CCL2, CCL3, CCL4, CCL5, CXCL9, and CXCL10, with significant elevation in SF vs PB for CXCL10. The subset expressing CXCR4 positively correlated with TNF-α, bone resorption marker, and rheumatoid factor, and it was reduced in patients treated with disease-modifying antirheumatic drugs. The CCR4^+^ subset showed a significant negative trend during anti-TNF treatment. CCL2, CCL5, and CXCL10 had similar osteoclastogenic effects, with CCL5 showing the greatest chemotactic action on OCPs.

**Conclusions:**

In our study, we identified distinct effects of selected chemokines on stimulation of OCP mobilization, tissue homing, and maturation. Novel insights into migratory behaviors and functional properties of circulatory OCPs in response to chemotactic signals could open ways to new therapeutic targets in RA.

**Electronic supplementary material:**

The online version of this article (doi:10.1186/s13075-017-1337-6) contains supplementary material, which is available to authorized users.

## Background

Rheumatoid arthritis (RA) is a systemic inflammatory disease manifesting predominantly as chronic affection of multiple joints and osteodestruction, and involving complex interactions between the immune system, extracellular matrix, and bone cells [1]. Severe clinical symptoms and the chronic course of the disease, underpinned by persistent inflammation, progressive joint destruction, and constant pain, require prolonged drug use and result, despite treatment, in significant skeletal deformities and global disability [2]. With a prevalence of around 1%, RA is one of the most common autoimmune diseases in the human population and an important contributor to public health burden, lower quality of life, and reduced life expectancy. Although numerous studies have been focused on the pathogenesis of RA, especially disease etiology and onset, many questions remain unanswered, specifically the underlying causes of perpetuation of chronic inflammation and sustained renewal of bone-resorbing cells [3, 4].

Osteoclasts (OCs) are multinucleated cells with the unique ability to resorb bone. They develop by fusion of hematopoietic precursors belonging to the monocyte/macrophage lineage. OCs express receptors for two crucial regulators of their differentiation and maturation: macrophage colony-stimulating factor (M-CSF) and receptor activator of nuclear factor- κB ligand (RANKL, CD254). The receptor for colony-stimulating factor-1 (cFms, CD115, M-CSF receptor) appears at early developmental stages of the myeloid lineage, whereas receptor activator of nuclear factor-κB (RANK; CD265) is expressed on committed osteoclast progenitors (OCPs) and is crucial for OC survival and activation [5–7]. Physiologically, the main source of RANKL is osteoblast lineage cells, but RANKL has also been shown to be expressed by activated T lymphocytes, mature B lymphocytes, natural killer cells, and synoviocytes in inflammatory conditions. Furthermore, elevated levels of a wide range of proinflammatory cytokines (interleukin-1 [IL-1], IL-6, IL-15, IL-17, IL-18, IL-21, IL-22, IL-23, tumor necrosis factor-α [TNF-α]) and chemokines (CC chemokine ligand 2 [CCL2], CCL3, CCL4, CCL5) act in synergy with M-CSF/RANK signals to promote osteoclastogenesis [8, 9].

Because the life span of an individual OC is only a couple of weeks, OCs must be constantly replenished to sustain pathological bone resorption in the chronic course of RA [3, 10, 11]. The peripheral blood (PB) monocyte pool, originating from myeloid bone marrow progenitors, contains 1–2% of cells capable of differentiating in vitro into fully functional OCs in the presence of M-CSF and RANKL [3, 12]. Owing to monocyte/macrophage lineage heterogeneity and few consensual specific markers of human OCPs, researchers have used various monocyte markers in an attempt to better characterize this osteoclastogenic subpopulation in PB. On the basis of previous work of several groups, including our own, lymphoid marker-negative (CD3, CD19, CD56), CD11b and CD14 double-positive (DP) subpopulation of peripheral blood mononuclear cells (PBMCs) were identified as significantly OCP-enriched in patients with RA and patients with psoriatic arthritis (PsA) [3]. Furthermore, it has been proposed that the erosive phenotype of various forms of inflammatory arthritides could be a result of the plasticity of these hematopoietic monocytic progenitors, depending on the cytokine and chemokine milieu in the bone marrow, circulation, and synovium [13].

Chemokines are known to orchestrate the recruitment of immune cells in RA and other inflammatory rheumatic diseases [14]. They may also be involved in OCP trafficking because cells at various stages of OC lineage express chemokine receptors (CC chemokine receptor 1 [CCR1] to CCR4, CXC chemokine receptor 3 [CXCR3], CXCR4, or CX3C chemokine receptor 1) [15–18]. However, the contribution of chemotactic signals to homing of OCPs and to their differentiation into mature OCs has not been fully elucidated [3]. The aim of our present study was to define the chemokine receptor profile of peripheral OCPs specifically induced by arthritis as well as the susceptibility of this cell subset to chemotactic signals. Raised levels of chemokines and chemotactic gradient between plasma and synovial fluid (SF) seem to contribute not only to increased migration of OCPs but also to their enhanced differentiation. Thus, immunophenotyping of chemokine receptors as well as measurement of respective chemokine levels may represent useful parameters to assess arthritis severity, disease progression, or response to therapy.

## Methods

### Patients

Patients with arthritis (RA, *n* = 129; PsA, *n* = 53) admitted to Clinical Hospital Center “Sestre Milosrdnice”, Clinical Hospital “Sveti Duh”, and Clinical Hospital Center “Zagreb” were included in the study after approval was obtained from the ethics committees of those institutions and of the University of Zagreb School of Medicine, and informed consent was received from patients. Control subjects (CTRL; *n* = 110) were admitted to the same institutions for noninflammatory etiology and had no previous history of arthritic or autoimmune diseases. A rheumatology specialist established the diagnosis of RA on the basis of American College of Rheumatology 2010 criteria, whereas PsA was diagnosed according to the Moll and Wright criteria [19, 20]. PB samples were obtained from patients with RA and patients with PsA during routine clinical assessments and processed for analysis. The following data were also collected from patients with arthritis: disease duration (years), patient and physician assessment of disease activity (visual analogue scale [VAS]), patient assessment of pain and fatigue (VAS), tender joint count (TJC; out of 28 joints), swollen joint count (SJC; out of 28 joints), erythrocyte sedimentation rate (ESR), C-reactive protein (CRP) level, Disease Activity Score in 28 joints (DAS28) calculated with ESR, Clinical Disease Activity Index (CDAI), treatment received (nonsteroidal anti-inflammatory drugs [NSAIDs], disease-modifying antirheumatic drugs [DMARDs], corticosteroids, and/or biological therapy), levels of rheumatoid factor (RF; determined by turbidimetry), and anticitrullinated protein antibodies (ACPA; determined by enzyme-linked immunosorbent assay [ELISA]). For some analyses, patients with RA were grouped on the basis of their DAS28 into severe (DAS28 > 5.1) and low/moderate cases (DAS28 2.6–5.1) [21–23]. A cohort of patients with arthritis (*n* = 24) assigned for therapeutic arthrocentesis of the affected knee was included to obtain SF samples. A small subset of patients (*n* = 9) scheduled for biological treatment with TNF inhibitors (etanercept 50 mg weekly or adalimumab 40 mg every 2 weeks) was followed by taking PB samples immediately before and at 2, 4, and 6 months after onset of therapy.

### Isolation of mononuclear cells

Blood samples were obtained between 9:30 and 11:30 a.m. PBMCs and plasma were separated from PB by centrifugation (20 minutes at 600 × *g*) with Histopaque (Sigma-Aldrich, St. Louis, MO, USA). Aspirated SF samples were separated by sequential centrifugation (5 minutes at 100 × *g*) into acellular SF supernatants and SF-derived cells (synovial fluid mononuclear cells [SFMCs]). Plasma and SF samples were stored at −20 °C until a cytometric bead array (CBA) or ELISA was performed, whereas PBMCs and SFMCs were immediately used for flow cytometric phenotyping, RNA extraction, cell sorting, osteoclastogenic cultures, and migration assays.

### Flow cytometry

Phenotype characterization of OCPs from PB was performed using an Invitrogen Attune flow cytometer (Thermo Fisher Scientific, Waltham, MA, USA) and analyzed using FlowJo software (FlowJo, LLC, Ashland, OR, USA). Cells were counted in a hemocytometer by trypan blue exclusion and labeled using a mix of commercially available monoclonal antibodies against lymphoid lineage markers (anti-CD3 fluorescein isothiocyanate [FITC] for T lymphocytes, anti-CD19 FITC for B lymphocytes, and anti-CD56 FITC for natural killer cells), myeloid lineage markers (anti-CD11b phycoerythrin [PE]-cyanine 7 [Cy7] and anti-CD14 peridinin-chlorophyll-cyanine 5.5), receptors for OC differentiation factors (receptor for M-CSF [biotinylated anti-CD115], RANK [anti-RANK PE]), and several chemokine receptors (anti-CCR1 allophycocyanin [APC], anti-CCR2 PE, anti-CCR4 APC, anti-CXCR3 APC, anti-CXCR4 PE) (all from eBioscience, San Diego, CA, USA). Suspensions were incubated on ice for 40 minutes, followed by washing in staining medium (PBS/2% FBS). As a second step (for biotinylated anti-CD115), cells were stained with streptavidin coupled to APC-eFluor 780 (Thermo Fisher Scientific) on ice for 40 minutes, followed by washing in staining medium, and afterward they were resuspended in staining medium for analysis. OCPs were defined as part of the monocytic subpopulation bearing the CD3^−^CD19^−^CD56^−^CD11b^+^CD14^+^ phenotype [3, 12]. The subpopulation was then analyzed for expression of CD115 and RANK as well as for subsequent chemokine receptor expression (CCR1, CCR2, CCR4, CXCR3, and CXCR4). Gates for the analysis were defined using unlabeled cells and "fluorescence minus one control".

### Cell sorting

For fluorescence-activated cell sorting (FACS), PBMCs of CTRL and RA samples were labeled using lymphoid lineage markers (anti-CD3 FITC for T lymphocytes, anti-CD19 FITC for B lymphocytes, anti-CD56 FITC for natural killer cells) and myeloid lineage markers (anti-CD11b APC and anti-CD14 PE-Cy7) and sorted using a BD FACSAria I cell sorter (BD Biosciences, Franklin Lakes, NJ, USA). Briefly, labeled cells were acquired at a speed of approximately 5000 cells/second, and singlets were delineated from the total cell population depicted on forward vs side scatter plots, followed by plotting lymphoid markers vs CD11b. Subsequently, lymphoid marker-negative cells, intermediately positive for CD11b, were gated for CD14 expression. A defined OCP population (CD3^−^CD19^−^CD56^−^CD11b^+^CD14^+^) was sorted in 2-ml collection tubes containing α-minimal essential medium (α-MEM)/20% FBS and used for osteoclastogenic cultures. Sorting purity was determined by reanalysis of fractioned populations and was consistently greater than 99%.

### Osteoclast differentiation from sorted peripheral blood mononuclear cells

Sorted OCPs were plated at a density of 4 × 10^4^ cells per well in 96-well plates in α-MEM/10% FBS. For the first 3 days of cell culture, 60 ng/ml recombinant human (rh)M-CSF (R&D Systems, Minneapolis, MN, USA) was added to stimulate cell proliferation. The next 8–11 days, the cell culture medium was supplemented with 100 ng/ml rhRANKL (R&D Systems) and 30 ng/ml rhM-CSF to stimulate differentiation into mature OCs. At days 11–13 of culture, OCs were enumerated by staining for tartrate-resistant acid phosphatase (TRAP).

### Migration assay

PBMCs were plated at a density of 10 × 10^4^ per well in 48-well plates in α-MEM/10% FBS and cultured overnight with 35 ng/ml M-CSF. Nonadherent cells enriched in OCPs were harvested for further analyses as previously described [12]. Considering the heterogeneity of the PB monocyte/macrophage lineage, the nonadherent fraction obtained after short-term incubation was comprised of a mixture of lymphocytes and immature monocytes. Lymphocytes were mostly removed by subsequent media changes.

A portion of nonadherent cells was used in osteoclastogenic culture additionally treated with chemokines. These cells were plated at a density of 2.5 × 10^4^ cells per well in 96-well plates in α-MEM/10% FBS supplemented by 100 ng/ml rhRANKL and 30 ng/ml rhM-CSF, with the addition of 40 ng/ml CCL2, 10 ng/ml CCL5, 20 ng/ml CXC chemokine ligand 10 (CXCL10) or 50 ng/ml CXCL12 (PeproTech, Rocky Hill, NJ, USA). Assays were performed on PB RA samples (*n* = 6–12 per chemokine treatment). After 10–11 days, cells were stained with TRAP for OC enumeration.

The remainder of nonadherent cells was used for the Transwell culture system. The cells were plated at a density of 5 × 10^4^ cells per well in 48-well plates in α-MEM/10% FBS with 100 ng/ml RANKL and 30 ng/ml M-CSF. At day 2 of culture, adherent cells from each sample were harvested and seeded into the upper chambers of 24-well Costar Transwell plates (8.0-μm pore size; Corning Inc., Corning, NY, USA) at a density of 10^4^ cells/well in 100 μl of α-MEM/10% FBS, and the lower chambers were filled with 40 ng/ml CCL2, 10 ng/ml CCL5, 20 ng/ml CXCL10, or 50 ng/ml CXCL12 (PeproTech) in 500 μl of α-MEM/10% FBS. After 3-h incubation at 37 °C with 5% CO_2_, the upper surface of the membrane was carefully washed with PBS, and the remaining cells were removed with a cotton wool swab. The cells that had migrated to the bottom side of the Transwell membrane inserts were fixed with 4% paraformaldehyde and stained with 4′,6-diamidino-2-phenylindole (DAPI). The migrated cells were counted (two wells per group, five central fields per Transwell) at × 200 magnification using a fluorescence microscope (Axiovert 200; Carl Zeiss AG, Oberkochen, Germany).

The cells that had migrated through the Transwell membrane to the lower chamber of the 24-well plate were cultured for the next 8–9 days in α-MEM/10% FBS supplemented with 100 ng/ml RANKL and 30 ng/ml M-CSF. At the end of the culture period, plates were stained for TRAP activity to determine the number of differentiated OCs.

### Detection of osteoclasts

The number of OCs differentiated from PBMCs or the sorted OCP-enriched population was determined by TRAP staining at the culture endpoint (days 10–13). Osteoclastogenic cultures were grown as previously described. Staining for TRAP was performed using a commercially available set of chemicals according to the manufacturer’s instructions (leukocyte acid phosphatase kit; Sigma-Aldrich). OCs were identified and enumerated as TRAP^+^ cells with two or more nuclei per cell at × 200 magnification by light microscopy using the Axiovert 200 fluorescence microscope.

Vitronectin receptor (VNR) expression and bone resorption assays were performed in parallel with TRAP staining to confirm the identity of OCs. For VNR detection, osteoclastogenic cultures were grown as previously described. At culture days 14–16, cells were fixed in 4% paraformaldehyde for 10 minutes and blocked for 30 minutes in 2% bovine serum albumin/PBS. Monoclonal biotinylated CD51/CD61 (integrin α_v_β_3_) antibody (1:80; eBioscience) was incubated for 2 h at room temperature. As a second-step reagent, streptavidin coupled to FITC was incubated for 1 h at room temperature. Cell nuclei were counterstained with DAPI. VNR expression was visualized at × 200 magnification using the Axiovert 200 fluorescence microscope.

For bone resorption assays, cells were plated over the bovine cortical bone slices using the same osteoclastogenic culture conditions as previously described. At culture days 16–18, cells were fixed in 4% paraformaldehyde for 10 minutes at room temperature. Following TRAP staining using a commercially available set of chemicals (leukocyte acid phosphatase kit; Sigma-Aldrich), cells were removed from bone slices by sonication in 0.25 M NH_4_OH for 5 minutes. Bone slices were then stained with 1% toluidine blue in 1% borax buffer for 2 minutes to visualize resorption pits at × 200 magnification using an Axiovert 200 light microscope.

### Cytometric bead array

CBA was performed using the Human Chemokine CBA Kit (BD Biosciences, Sans Jose, CA, USA) to assess the levels of CCL2, CCL3, CCL4, CCL5, CXCL9, and CXCL10 in PB and SF samples. Plasma and SF supernatants were each diluted 1:4 using the supplied dilution buffer and incubated with capture beads. Each capture bead was coated with a capture antibody specific for each soluble protein and could be identified by its distinct fluorescence signature in two fluorescence channels on the flow cytometer (APC vs APC-Cy7). The samples were then washed and incubated with the detection reagent. The concentration of the bound analyte was determined by the intensity of the PE fluorescence, depending on the amount of bound PE-conjugated antibodies. After another washing step, complexes of capture beads, analytes, and detection reagents were analyzed using Invitrogen Attune flow cytometer. Concentrations of chemokines were determined using FCAP Array version 3.0 software (BD Biosciences), based on standard curves generated by data acquired from provided protein standards.

### Gene expression analysis by quantitative polymerase chain reaction

Total RNA was extracted from PBMCs using Invitrogen TRIzol reagent (Life Technologies, Carlsbad, CA, USA). For polymerase chain reaction (PCR) amplification, 1 μg of total RNA was converted to complementary DNA (cDNA) by reverse transcriptase (Applied Biosystems, Foster City, CA, USA) and stored at −20 °C until further analysis. The amount of cDNA corresponding to 20 ng of reverse-transcribed RNA was amplified by quantitative polymerase chain reaction (qPCR) in an ABI Prism 7500 sequence detection system (Applied Biosystems). Gene expression of CCL2 (hs00234140_m1), CCL3 (hs00234142_m1), CCL4 (hs99999148_m1), CCL5 (hs00982282_m1), and CXCL12 (hs02668440_m1) was analyzed using commercially available TaqMan assays (Applied Biosystems). Each reaction was performed in duplicate in a 25-μl reaction volume. The relative quantities were calculated using the standard curve designed from six serial dilutions of the calibrator sample (CTRL PBMCs). According to the standard curve, the relative amounts of RNA for target genes were calculated as the ratio of the quantity of the target gene normalized to *GAPDH* (glyceraldehyde 3-phosphate dehydrogenase) as the endogenous control.

### ELISA

Plasma concentrations of TNF-α (Quantikine HS ELISA kit; R&D Systems), CXCL12 (Quantikine Immunoassay; R&D Systems), and carboxy-terminal telopeptide of type I collagen (CTX; Immunodiagnostic Systems, The Boldons, UK), as well as SF concentrations of RANKL (Quantikine Immunoassay; R&D Systems), were determined in accordance with the manufacturers’ instructions. Briefly, samples were incubated for 2–3 h at room temperature on monoclonal antibody-precoated plates, washed three times, and incubated for the next 2 h with HRP-conjugated (CXCL12, RANKL) or alkaline phosphatase-conjugated (TNF-α, CTX) specific antibodies. After another three washes, the reactions were visualized with substrate solution (tetramethylbenzidine) (CXCL12, RANKL) or substrate and amplifier solutions (nicotinamide adenine dinucleotide phosphate and amplifier enzymes) (TNF-α, CTX) and then arrested with hydrochloric or sulfuric acid, respectively. Optical density was determined within 15 minutes on a microplate reader (Bio-Rad Laboratories, Hercules, CA, USA) set to an excitation wavelength of 450 nm (CXCL12, RANKL) or 490 nm (TNF-α, CTX).

### Statistical analysis

Data were presented as median with IQR for continuous variables (including clinical scores, laboratory measures, protein concentrations, cell numbers and frequencies) or categorized for categorical variables (including sex, DAS28 subgroup, and treatment). Statistical analysis of the group differences was performed using a nonparametric Mann-Whitney *U* test (for group-to-group comparisons) or the Kruskal-Wallis test followed by the Mann-Whitney *U* test (for multiple comparisons). Correlations were done using rank correlation and Spearman’s coefficient ρ with 95% CI. Trend analysis was performed on population frequencies during anti-TNF therapy by using one-way analysis of variance (ANOVA) with polynomial contrast type and testing for linear trends. Multiple logistic regression analysis was performed with the frequency of the PB OCP cells (CD3^−^CD19^−^CD56^−^CD11b^+^CD14^+^) as the dependent variable. Parameters were taken into consideration as independent variables if comparative analysis between CTRL and RA values showed a significant difference. An extensive search was then performed to see which three of the significantly different independent variables resulted in maximal adjusted *R* ^2^ values, with pairwise exclusion of missing values in the analysis. Statistical analyses were performed using MedCalc for Windows version 9.4.2.0 software (MedCalc Software, Ostend, Belgium). For all experiments, the α-level was set at 0.05.

## Results

### Patients with rheumatoid arthritis have highly active disease with elevated inflammatory, osteoresorptive, and autoimmune indicators

The demographic and clinical characteristics showed that the majority of patients with RA included in the study had a chronic course of active disease (median DAS28 5.97; IQR 4.99–6.71) (Table 1) with multiple joints affected and enhanced osteoresorption (median CTX 0.38 [IQR 0.26–0.49] pg/ml in RA vs 0.28 [IQR 0.21–0.32] pg/ml in CTRL). The majority of patients were seropositive with elevated inflammatory indicators (ESR and CRP). Although patients were actively treated (by NSAID, DMARD, glucocorticoid, or biologic therapy), DAS28 and other clinical parameters (SJC, TJC, CDAI) remained high, indicating poor regulation and disease progression. Compared with CTRL subjects, there were no differences between groups in terms of age (median age 65 [IQR 48–68] years for CTRL vs 65 [IQR 51–74] years for RA) or sex (male/female ratio 21/89 for CTRL vs 15/114 for RA).

**Table 1:** Demographic and clinical characteristics of patients with rheumatoid arthritis and psoriatic arthritis

	RA	PsA
Age, years	65 [51–74]	56 [44–74]
Sex, male/female	15/114	24/29
DAS28-ESR	5.97 [4.99–6.71]	5.50 [4.05–6.78]
DAS28-ESR 2.6–5.1/DAS28-ESR >5.1, %	25.0%/75.0%	41.8%/58.2%
CDAI	37.0 [24.0–44.1]	33.6 [14.0–52.1]
Disease duration, years	18.5 [9.0–29.0]	14.0 [8.0–30.0]
ESR, mm/h	27.5 [19.0–39.5]	14.5 [4.0–46.5]
CRP, mg/L	11.9 [4.8–24.5]	2.7 [0.7–29.1]
RF, IU/L	79.7 [15.0–239.0]	N/A
ACPA, EU/L	68.7 [2.2–270.8]	N/A
Tender joint count (out of 28)	15 [8–22]	17 [3–26]
Swollen joint count (out of 28)	7 [2–12]	6 [0–14]
Disease activity, cm, physician’s VAS assessment	7.0 [5.5–8.8]	6.4 [4.7–9.0]
Disease activity, cm, patient’s VAS assessment	7.0 [5.0–8.8]	6.9 [4.9–8.2]

*Abbreviations: RA* Rheumatoid arthritis, *PsA* Psoriatic arthritis, *ESR* Erythrocyte sedimentation rate, *CRP* C-reactive protein, *CDAI* Clinical Disease Activity Index, *DAS28-ESR* Disease Activity Score in 28 joints based on erythrocyte sedimentation rate, *VAS* Visual analogue scale, *RF* Rheumatoid factor, *ACPA* Anticitrullinated protein antibodies, *EU * ELISA units, *N/A* Not applicable

Values are presented as median with IQR, except DAS28 and sex categories

**Fig. 1:**
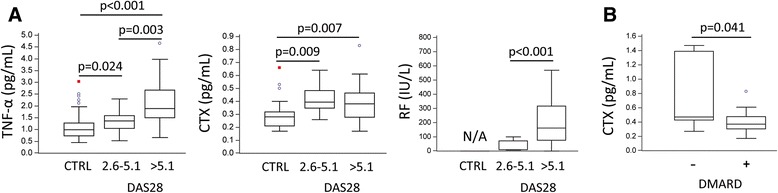
Elevated indicators of inflammatory activity, osteoresorption, and autoimmunity in rheumatoid arthritis (RA). **a** Plasma levels of tumor necrosis factor-α (TNF-α), carboxy-terminal telopeptide of type I collagen (CTX), and rheumatoid factor (RF) in control subjects (CTRL) and patients with RA, categorized by Disease Activity Score in 28 joints (DAS28) as low/moderate RA (DAS28 2.6–5.1) and severe RA (DAS28 > 5.1). **b** Plasma CTX levels with regard to disease-modifying antirheumatic drug (DMARD) therapy in patients with RA. Values are presented as medians (*middle lines*), with *boxes* representing IQR, *whiskers* representing 1.5 times the IQR, and *squares* or *circles* representing outliers. Group-to-group comparisons were performed using a nonparametric Mann-Whitney *U* test, and *p* values <0.05 are shown. *N/A* Not applicable

To distinguish patients with RA on the basis of disease activity and to set the frame for further comparisons with laboratory and experimental data, we grouped disease parameters into variables reflecting the intensity of inflammation (CRP, ESR, TNF-α), joint destruction/bone resorption (CTX, SJC/TJC, CDAI), and autoimmune reaction (RF, ACPA), and we analyzed them with respect to DAS28 categories (Fig. 1a). Levels of both TNF-α and RF were significantly higher in severe cases (DAS28 > 5.1) than in CTRL cases and low/moderate cases (DAS28 2.6–5.1). As a marker of bone resorption rate, plasma CTX did not increase with disease severity, which can be explained by a number of factors besides RA that affect bone turnover (including patient age, glucocorticoid use, and immobility) [24]. In addition, with regard to treatment modalities, lower CTX values were observed in DMARD-treated patients (Fig. 1b).

### Circulating osteoclast progenitor cells from arthritic patients have distinct functional and phenotypic features

**Fig. 2:**
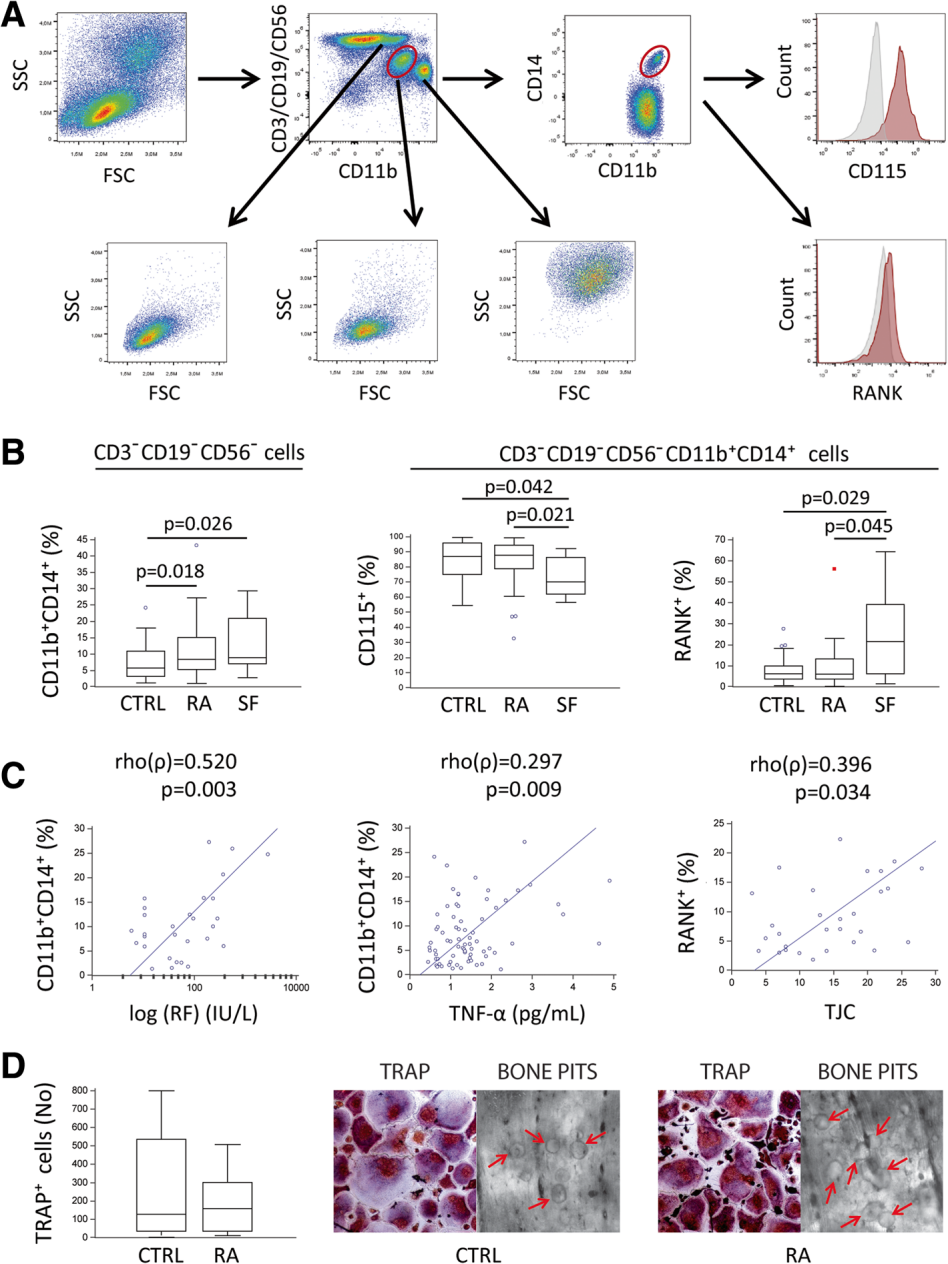
Expanded osteoclast progenitor (OCP) population correlates with indicators of disease activity in rheumatoid arthritis (RA). **a** Peripheral blood mononuclear cells were analyzed by flow cytometry using the gating strategy for OCPs as nonlymphoid (CD3^−^CD19^−^CD56^−^) cells double-positive for monocyte markers CD11b and CD14, and they were further assessed for the expression of macrophage colony-stimulating factor receptor (CD115) and receptor activator of nuclear factor-κB (RANK). **b** Proportion of OCPs, and CD115^+^ and RANK^+^ subsets in peripheral blood of control (CTRL) subjects and patients with RA, as well as RA synovial fluid (SF) samples. Gates for the analysis were defined using unlabeled cells and "fluorescence minus one control". **c** Association of the proportion of circulating OCPs with plasma levels of rheumatoid factor (RF) and tumor necrosis factor-α (TNF-α), as well as association of the proportion of RANK^+^ OCPs with tender joint count (TJC). **d** Quantification, morphology, and bone-resorbing activity of osteoclasts (OCs) differentiated from sorted peripheral blood OCPs in RA and CTRL samples. Osteoclastogenic potential of circulating OCPs was evaluated in vitro by stimulating sorted CD3^−^CD19^−^CD56^−^CD11b^+^CD14^+^ cells with macrophage colony-stimulating factor (30 ng/ml) and RANK ligand (100 ng/ml). Multinuclear cells positive for tartrate-resistant acid phosphatase (TRAP) were counted as mature OCs. Bone-resorbing activity was confirmed for sorted cells by pit formation assay under the same culture conditions. TRAP-stained mature OCs and bovine cortical bone slices from CTRL and RA cultures were scanned using light microscopy at × 200 magnification. *Red arrows* indicate bone resortion pits, formed by active mature osteoclasts. Values are presented as medians (*middle lines*), with *boxes* representing IQR, *whiskers* representing 1.5 times the IQR, and *squares* or *circles* representing outliers. Group-to-group comparisons were performed using a nonparametric Mann-Whitney *U* test, whereas correlations were done using rank correlation and Spearman’s coefficient ρ. *p* Values <0.05 are shown

On the basis of our previous findings [3, 12], PBMCs of CTRL subjects and subjects with arthritis were analyzed by FACS to determine the frequency of OCP populations using the gating strategy presented in Fig. 2a. Briefly, total PBMCs, depicted on forward vs side scatter plots, were analyzed by plotting lymphoid markers vs CD11b. Subsequently, a population of lymphoid-negative cells (CD3^−^CD19^−^CD56^−^) expressing CD11b (CD11b^+^) was confirmed by size and granularity to represent the monocyte subpopulation and then further dissected for the expression of CD14, CD115, and RANK. Lymphoid-negative cells that highly expressed CD11b (CD11b^++^) correspond to granulocytes, whereas lymphoid-positive cells negative for CD11b correspond to lymphocytes. In addition to PBMCs, SFMCs were analyzed using a similar gating strategy to determine specific systemic-to-local differences between myeloid cell subsets.

We presumed that the inclusion of an RA cohort with a significant proportion of highly active disease (sustained inflammation and joint destruction) would allow for a clearer detection of distinct OCP subpopulations induced by arthritis. Therefore, we first determined differences in PB of CTRL and RA samples. The DP (CD11b^+^CD14^+^) subset of PB monocytes was moderately expanded in patients with RA compared with CTRL subjects (Fig. 2b). In RA, the proportion of these cells was positively associated with plasma levels of TNF-α and RF (Fig. 2c) but not with the level of CTX (not shown). The DP PB population possesses osteoclastogenic potential because we were able to obtain fully functional bone-resorbing OCs from sorted cells under stimulation by M-CSF and RANKL (Fig. 2d). However, this population generated numbers of differentiated TRAP^+^ OCs similar to those of CTRL subjects when seeded at the same densities. In line with the latter finding, the frequency of PB DP cells expressing corresponding receptors—CD115 (M-CSF receptor) and RANK (RANKL receptor)—was comparable between CTRL subjects and patients with RA, indicating similar responsiveness to the respective ligands (Fig. 2b).

We further assessed differences between the circulatory and synovial compartments of patients with RA and found that the proportion of DP monocytes was comparable between PB and SF samples (Fig. 2b). Because it was not feasible to obtain appropriate CTRL SF samples (owing to the limited amount and low cellularity of SFs in healthy synovial joints, as well as ethical unacceptability), we could not compare the SFMC profile between CTRL subjects and patients with RA. However, the proportion of DP SFMCs in RA compared with DP PBMCs in CTRL subjects was statistically higher, indicating a general increase of the DP population associated with RA. Expression of CD115 was lower in DP SFMCs than in the PBMC subset of patients with arthritis, which can be explained by enhanced shedding of membrane receptors for proinflammatory mediators seen as a negative feedback loop in inflamed tissues [25]. Nevertheless, the population of DP monocytes expressing RANK, considered to represent progenitors committed to OC lineage [3], was enlarged in the synovial vs the circulatory compartment, showing a positive correlation with TJC (Fig. 2c).

At this point, we also aimed to assess whether observed changes were specifically associated with RA compared with other forms of arthritis, as well as to analyze another group of SFMC samples (not possible to obtain from CTRL subjects). We included patients with PsA with affected peripheral joints; they resemble RA by having osteodestructive chronic inflammatory polyarthritis but are almost always seronegative with distinct pathogenic and clinical features. In general, the frequency of osteoclastogenic subpopulations followed a pattern in patients with PsA similar to that in patients with RA. Although the increase in proportion of PB DP monocytes did not reach significance, the proportion of these cells expressing RANK was significantly larger than in CTRL subjects (Additional file 1: Figure S1a). In our previous study, we indicated that the RANKL-RANK axis may be specifically associated with PsA compared with RA pathogenesis [12]. However, the level of soluble RANKL was not significantly higher in PsA SF than in RA SF (*p* = 0.355), possibly owing to the small sample size (Additional file 1: Figure S1b).

### Circulating osteoclast progenitors expressing chemokine receptors are exposed to higher plasma chemokine levels

**Fig. 3:**
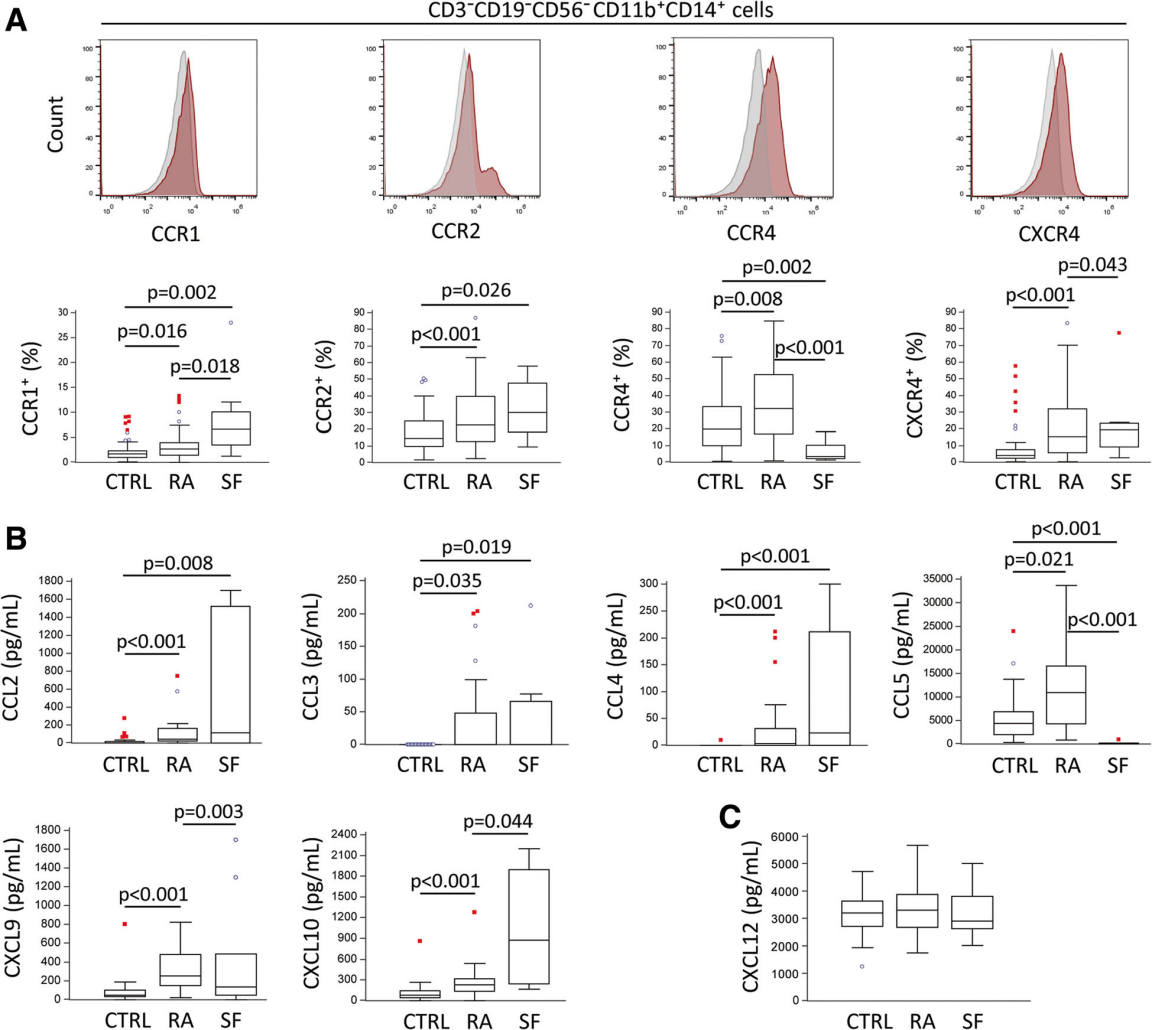
Increased expression of chemokine receptors by osteoclast progenitors and higher chemokine levels in plasma and synovial fluid (SF) of patients with rheumatoid arthritis (RA). **a** Flow cytometric analysis of chemokine receptors on osteoclast progenitors (CD3^−^CD19^−^CD56^−^CD11b^+^CD14^+^) in peripheral blood of control subjects (CTRL) and patients with rheumatoid arthritis (RA), as well as in RA SF samples. Gates for the analysis were defined using unlabeled cells and "fluorescence minus one control". **b** Levels of chemokine ligands in peripheral blood of CTRL subjects and patients with RA, as well as in RA SF samples, measured by a flow cytometry-based, predesigned cytometric bead array. **c** Levels of CXC chemokine ligand 12 (CXCL12) in peripheral blood of CTRL subjects and patients with RA, as well as in RA SF, measured by enzyme-linked immunosorbent assay. Values are presented as medians (*middle lines*), with *boxes* representing IQR, *whiskers* representing 1.5 times the IQR, and *squares* or *circles* representing outliers. Group-to-group comparisons were performed using a nonparametric Mann-Whitney *U* test, and *p* values <0.05 are shown. *CCR* CC chemokine receptor, *CXCR* CXC chemokine receptor, *CCL* CC chemokine ligand

After a functional confirmation of osteoclastogenic potential for CD3^−^CD19^−^CD56^−^CD11b^+^CD14^+^ cells, but without increases in the number of differentiated TRAP^+^ OCs under M-CSF/RANKL stimulation in patients with arthritis, we hypothesized that the enhanced OCP activity might be caused by the complementary proinflammatory/osteoclastogenic stimuli, namely chemokine signals [3]. Monocytes, which comprise OCPs, are known to express a variety of chemokine receptors, which, upon ligand binding, initiate multiple cellular responses, including migration, proliferation, differentiation, and activation [26]. Proportions of DP subpopulations in PB were higher for several chemokine receptors in patients with RA (CCR1, CCR2, CCR4, CXCR4) (Fig. 3a) and in patients with PsA (CCR1, CXCR4) (Additional file 1: Figure S1c). In addition, receptor expression for CCR1 and CXCR4 was significantly higher in SFMCs than in PBMCs of patients with RA. Only the monocyte expression of CCR4, with unique ligands (CCL17 and CCL22) compared with CCR1 and CCR2 [14], was significantly lower in SFMCs than in PBMCs of patients with RA.

Upregulation of the distinct set of chemokine receptors on OCPs suggested their high susceptibility to chemokine signals and prompted us to determine the chemokine ligand profile in plasma and SF of patients with RA. Using the flow cytometry-based, predesigned Human Chemokine CBA Kit, we detected higher levels of CCL2, CCL3, CCL4, CCL5, CXCL9, and CXCL10 in arthritis than in CTRL plasma (Fig. 3b). Moreover, CXCL10 was significantly higher in SF than in plasma of patients with RA, indicating a chemotactic gradient for cell migration from the circulation to the synovial compartment. The increase of CCL2, CCL3 and CCL4 levels in SF of patients with RA was significant compared with CTRL plasma, considered as the basal level. However, the difference was not significant compared with RA plasma, probably owing to the small number of SF samples and high variability (with part of the measurements below the detection limit of the CBA). Additionally, CXCL12 was measured by ELISA, with no difference found between groups (Fig. 3c). Indirectly, we confirmed that PBMCs were not the major source of chemokines by analyzing their gene expression using qPCR (Additional file 2: Figure S2). With the exception of CCL5, which was abundant at protein and gene expression levels, chemokines were barely detectable by qPCR, and there were no differences between groups, suggesting that they are produced by cells other than PBMCs and egress into the circulation to exert chemotactic effects.

### Chemokine receptor/ligand levels are associated with clinical variables and indicate biological significance for disease pathogenesis and progression

**Fig. 4:**
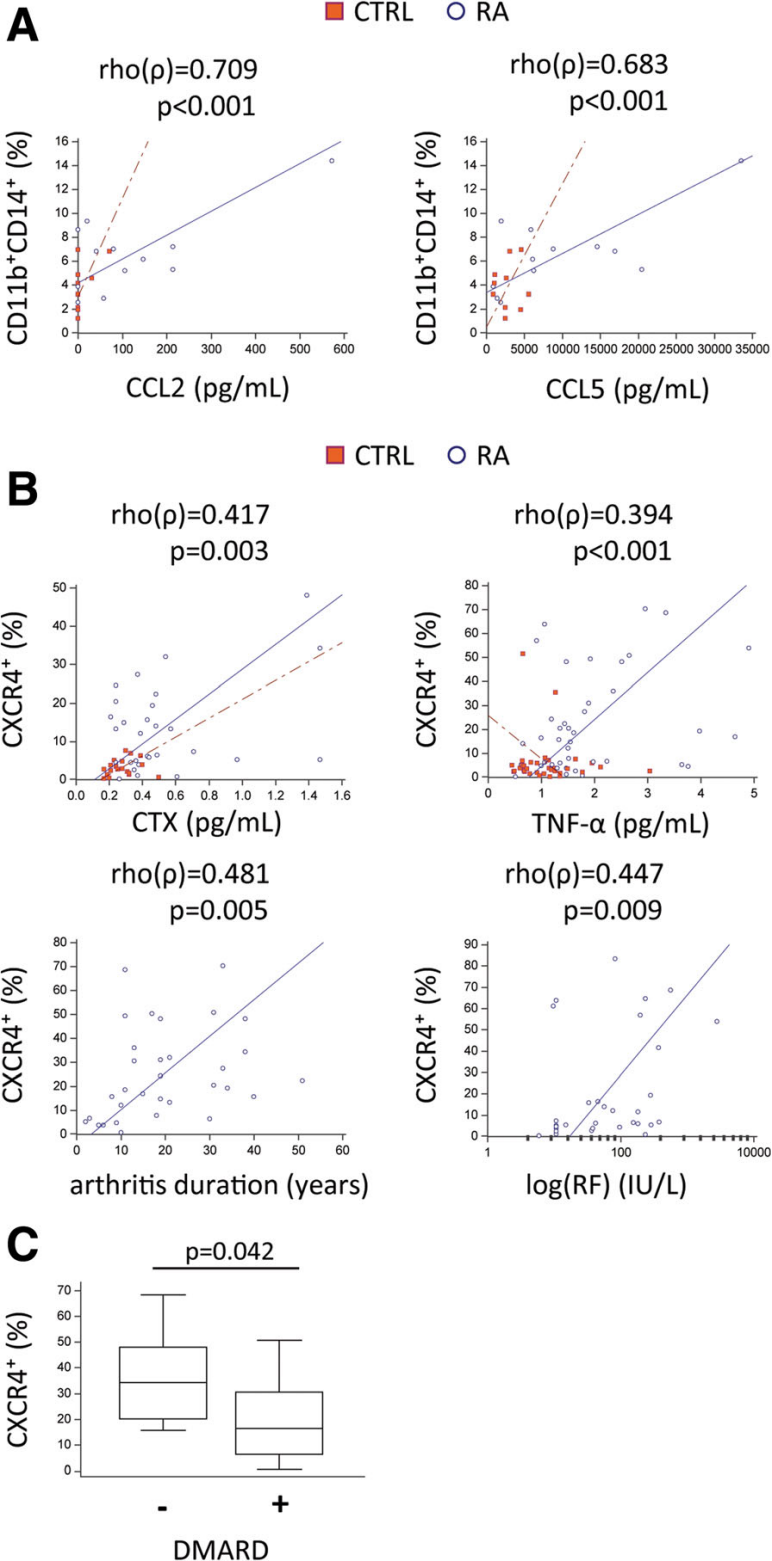
Association of peripheral blood osteoclast progenitor (OCP) frequency and proportions of subsets expressing chemokine receptors with chemokine levels and indicators of disease activity. **a** Association of peripheral blood OCP frequency (CD3^−^CD19^−^CD56^−^CD11b^+^CD14^+^) with plasma concentration of CC chemokine ligands (CCL2, CCL5) in control subjects (CTRL) and patients with rheumatoid arthritis (RA). **b** Association of CXC chemokine receptor 4 (CXCR4) expression in OCPs with arthritis duration, plasma levels of tumor necrosis factor-α (TNF-α), carboxy-terminal telopeptide of type I collagen (CTX), and rheumatoid factor (RF). **c** Proportions of CXCR4^+^ OCPs in patients with or without treatment with disease-modifying antirheumatic drugs (DMARDs). Association was established using rank correlation, and Spearman’s coefficient ρ and *p* values are shown for total data, including both CTRL and RA, whereas group trend lines are shown separately as *dashed lines* for CTRL and *solid lines* for RA. Group-to-group comparisons were performed using a nonparametric Mann-Whitney *U* test, and *p* values <0.05 are shown. Values are presented as medians (*middle lines*), with *boxes* representing IQR, *whiskers* representing 1.5 times the IQR, and *squares* or *circles* representing outliers

To this point, our data confirmed increased chemokine levels in patients with RA, which may be responsible for enhanced migration of OCPs expressing appropriate receptors. Therefore, we further assessed the association between the frequency of OCPs and their chemokine receptor expression with the level of proinflammatory mediators and clinical parameters of the disease. The proportion of PB DP monocytes was positively associated with plasma levels of CCL2 and CCL5 (Fig. 4a), and both ligands were positively associated with TNF-α (ρ = 0.549, *p* < 0.001 for CCL2; ρ = 0.554, *p* < 0.001 for CCL5). In addition, the proportion of DP subsets expressing CCR1 and CCR4 was significantly associated with plasma levels of corresponding chemokine ligands (ρ = 0.477, *p* = 0.006 for CCR1 with CCL3; ρ = 0.655, *p* < 0.001 for CCR4 with CCL2; ρ = 0.420, *p* = 0.010 for CCR4 with CCL3). Besides findings in patients with RA, the proportion of PB DP monocytes was positively associated with ESR in patients with PsA (ρ = 0.822, *p* < 0.001).

Among analyzed chemokine receptors, the DP subset expressing CXCR4 showed the most significant associations with inflammatory indicators and disease activity, including TNF-α concentration, duration of arthritis, autoantibody levels, and bone resorption (Fig. 4b). Moreover, the proportion of the DP subset expressing CXCR4 was significantly lower in patients treated with DMARD (Fig. 4c), whom we showed to also have a lower bone resorption rate (Fig. 1b). Therefore, we concluded that the highly osteoclastogenic DP population of PBMCs, especially the subset expressing CXCR4, may be used as an indicator of disease activity and response to therapy.

Finally, we assessed the contribution of analyzed data to the frequency of PB DP cells and found that multiple logistic regression was able to explain 79% of the variability of the DP subpopulation frequency (adjusted *R* ^2^ = 0.790) from CCL5, CXCL10, and CXCR4 values. These variables significantly predicted the frequency of DP monocytes (*p* < 0.001), adding to the prediction in the following order: CCL5 (β = 0.527, *p* < 0.0001), CXCL10 (β = 0.522, *p* < 0.0001), and CXCR4 (β = 0.284, *p* = 0.015).

### Chemokine receptor expression is significantly affected by anti-TNF therapy, indicating involvement of chemokine signals in anti-TNF response

Therapeutic targeting of proinflammatory mediators has been approved for arthritis treatment to suppress inflammatory responses as well as subsequent local and systemic bone loss. Our preliminary data on PsA samples (*n* = 4) showed a positive association between the proportion of PB DP monocytes and DAS28, as well as reduction of both variables along with the anti-TNF treatment in two of four patients [3].

**Fig. 5:**
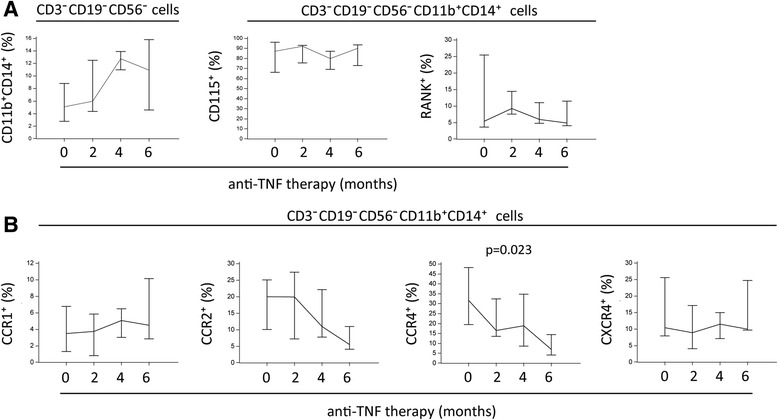
Alteration of chemokine receptor expression on circulatory osteoclast progenitors (OCPs) in follow-up of anti-TNF therapy. **a** Proportion of OCPs (CD3^−^CD19^−^CD56^−^CD11b^+^CD14^+^) and their subsets expressing macrophage colony-stimulating factor receptor (CD115) and receptor activator of nuclear factor-κB (RANK) in peripheral blood of patients with rheumatoid arthritis (RA) and patients with psoriatic arthritis (PsA) before (0) and 2, 4, and 6 months after induction of anti-TNF therapy (etanercept 50 mg weekly or adalimumab 40 mg every 2 weeks). **b** Subsets of OCPs expressing chemokine receptors with regard to duration of anti-TNF therapy in patients with RA and patients with PsA. Values are presented as median with IQR for each time point. Analysis for linear trend was performed using one-way analysis of variance with polynomial contrast type. *p* Values <0.05 are shown. *CCR* CC chemokine receptor, *CXCR* CXC chemokine receptor

In this study, the small cohort of patients (RA, *n* = 4; PsA, *n* = 5) receiving anti-TNF therapy was followed to 6 months after the initial application. PBMCs were analyzed as previously described for patients with RA and patients with PsA (Fig. 2a). The total DP population was not suppressed by the treatment, and neither were the subsets expressing receptors for crucial osteoclastogenic factors: CD115 and RANK (Fig. 5a). In general, individual values were highly variable even at the starting point, which may be due to the fact that included patients were already in the chronic phase of the disease, having had arthritis for more than 10 years and having previously been treated with different combinations of NSAIDs, glucocorticoids, and DMARDs. However, the subpopulation of DP monocytes expressing CCR4 showed significant dynamic changes in response to anti-TNF therapy, exhibiting a significant negative trend during the anti-TNF treatment in patients with RA and patients with PsA, offering the possibility of using it as an indicator of a good therapeutic response (Fig. 5b). We observed elevated plasma levels of major CCR4 ligands (CCL2, CCL3, CCL5) and a significant positive correlation of CCR4 expression with CCL2 and CCL3 in patients with RA. Therefore, the downregulation of CCR4 may be important to reducing the susceptibility of OCPs to the chemokine gradient.

### Functional in vitro confirmation of direct osteoclastogenic and chemotactic effect of chemokines on circulating osteoclast progenitors

**Fig. 6:**
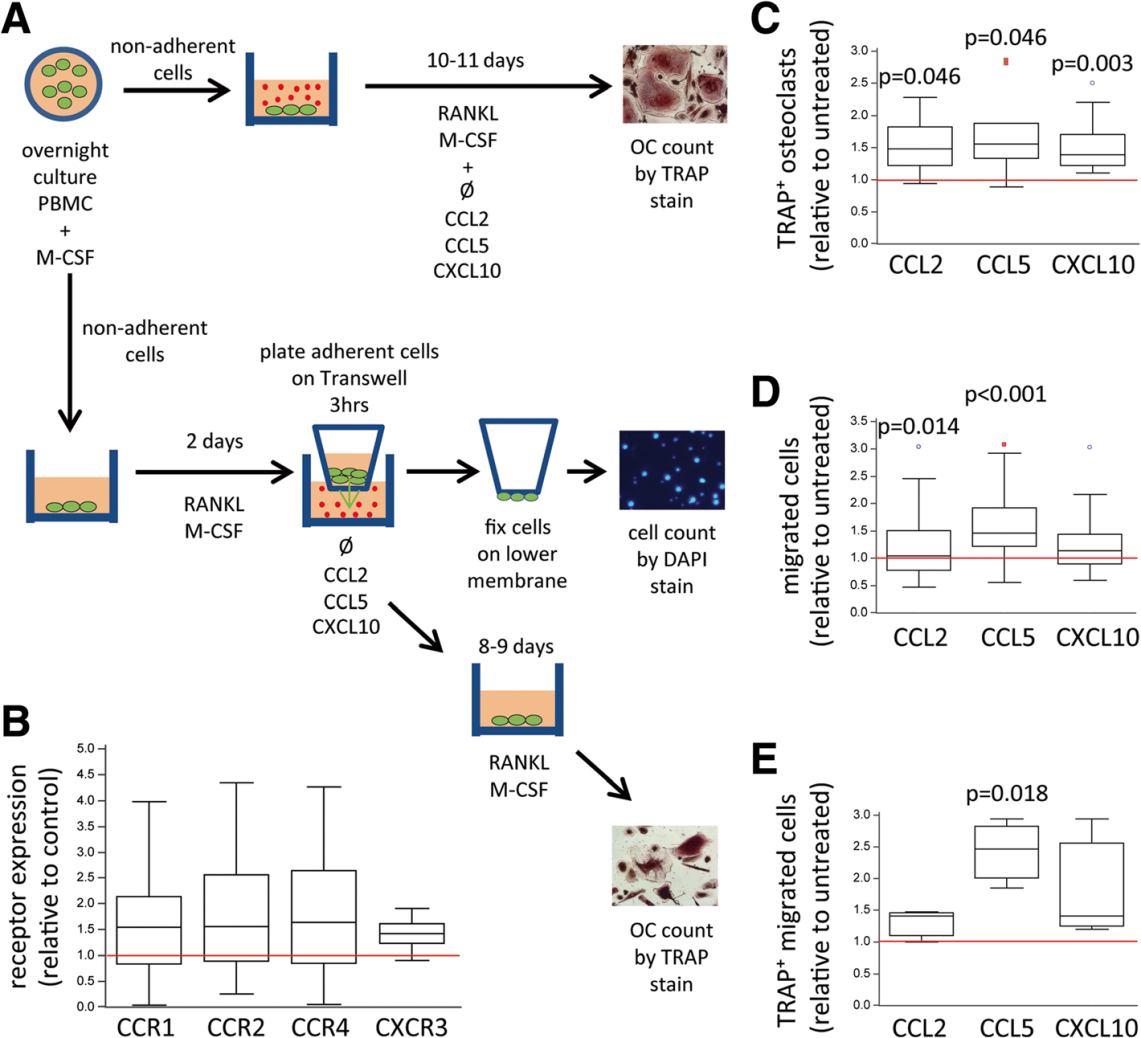
Stimulatory osteoclastogenic and chemotactic effects of chemokines on circulating osteoclast progenitors in rheumatoid arthritis. **a** Schematic presentation of experimental design. We evaluated chemokine osteoclastogenic and chemotactic effects on nonadherent cells harvested after overnight culturing of peripheral blood mononuclear cells (PBMCs) with 35 ng/ml macrophage colony-stimulating factor (M-CSF). For the osteoclastogenic assay, cells were further cultured with M-CSF (30 ng/ml) and receptor activator of nuclear factor-κB ligand (RANKL; 100 ng/ml), supplemented by CC chemokine ligand 2 (CCL2; 40 ng/ml), CCL5 (10 ng/ml), or CXC chemokine ligand 10 (CXCL10; 20 ng/ml). Multinuclear cells positive for tartrate-resistant acid phosphatase (TRAP) were counted as mature osteoclasts (OCs). For the migration assay, cells were first cultured for 2 days with M-CSF and RANKL, then transferred to a Transwell culture system, applying chemokine gradient (CCL2, CCL5, or CXCL10). Cells migrating through the Transwell membrane were enumerated by 4′,6-diamidino-2-phenylindole (DAPI) staining. Migrated cells were further differentiated by M-CSF and RANKL and assessed for the number of TRAP^+^ OCs. **b** Relative expression of chemokine receptors on osteoclast progenitors (CD3^−^CD19^−^CD56^−^CD11b^+^CD14^+^) in peripheral blood samples of patients with rheumatoid arthritis compared with the median value of control (healthy) subjects, as measured by flow cytometry. **c** Relative number of TRAP^+^ OCs generated by chemokine treatment compared with respective untreated osteoclastogenic culture (no chemokine). **d** Relative number of cells migrating by chemokine gradient through Transwell membrane compared with respective untreated migration assay (no chemokine). **e** Relative number of TRAP^+^ OCs generated from cells that had migrated to the lower chamber compared with respective untreated osteoclastogenic culture (no chemokine). Values are presented as medians (*middle lines*), with *boxes* representing IQR, *whiskers* representing 1.5 times the IQR, and *squares* or *circles* representing outliers. *Red horizontal lines* represent the relative value of the respective control. Group-to-group comparisons were performed using a nonparametric Mann-Whitney *U* test, and *p* values <0.05 are shown. *CCR* CC chemokine receptor, *CXCR* CXC chemokine receptor

Finally, we functionally tested the effects of chemokines on circulatory monocytes in vitro for their chemotactic and osteoclastogenic properties. PBMCs from individual samples of RA patients were first stimulated by M-CSF overnight, then nonadherent cells were harvested and split into two groups: One was grown further in osteoclastogenic culture conditions under chemokine treatment to assess direct osteoclastogenic effects, and the other was plated in a Transwell culture system to determine chemotaxis of OCPs toward the chemokine gradient (Fig. 6a). The latter migration assay was further analyzed for the number of attracted cells and for their osteoclastogenic potential. For these experiments, we selected CCL2, CCL5, and CXCL10 on the basis of receptor/ligand profiling in PB samples (Fig. 3). Besides higher CCR1, CCR2, and CCR4 (that bind CCL2 and CCL5), we found that CXCR3 (receptor for CXCL10) was also increased on OCPs in patients with RA (median 43.1% [IQR 33.3–54.2] in CTRL vs 61.5% [IQR 53.3–69.2] in RA, *p* = 0.009). CCL2 was taken as representative of a chemokine involved only in monocyte recruitment, whereas CXCL10 was taken as a chemokine that nonselectively attracts both myeloid and lymphoid lineages. In addition, we tested CCL5, which was found to be elevated in plasma but lower in SF, indicating its role in systemic activation rather than in joint-specific homing [27]. On the basis of CXCR4 expression (Fig. 3a), CXCL12 was also selected for in vitro migration assays, but in our culture conditions, the effects on osteoclastogenesis or chemotaxis were marginal or not observed, respectively (median ratio 1.31 [IQR 1.21–1.38] for the number of OCs in CXCL12-treated vs nontreated cultures, *p* = 0.106; ratio 0.97 [IQR 0.77–1.56] for the number of migrated cells in CXCL12-treated vs nontreated cultures, *p* = 1.000).

We expected great susceptibility of PB monocytes to chemokine signals in RA, considering their higher expression of chemokine receptors than CTRL (Fig. 6b). In general, CCL2, CCL5, and CXCL10 showed measurable osteoclastogenic as well as chemotactic effects. In the direct osteoclastogenic effect, all tested chemokines showed comparable potential (doses were optimized in accordance with the median effective dose provided by the manufacturer) and generated similar numbers of TRAP^+^ OCs (Fig. 6c). In addition, the identity of differentiated OCs was further confirmed by expression of VNR and bone-resorbing activity (Additional file 3: Figure S3). Interestingly, CCL5 showed the greatest chemotactic effects among tested chemokines by attracting the highest number of cells in the migration assay (Fig. 6d). In addition, these migrated cells possessed greater osteoclastogenic potential when stimulated by RANKL and M-CSF than untreated cells and cells attracted to CCL2 and CXCL10 gradient (Fig. 6e). These results imply that chemokine signals not only stimulate chemotaxis of OCPs but also act synergistically with RANKL and M-CSF to enhance their differentiation into mature OCs.

## Discussion

Our study provides evidence of the importance of chemokine signals to complement the canonical M-CSF/RANKL-mediated OC differentiation pathway by stimulation of OCP migration and maturation. Although genetic experiments in mice and primary human cell cultures have helped define the regulation of OC differentiation under physiological conditions, alternative pathways of OC stimulation, especially in patients with inflammatory conditions, are not yet clear. The complex environment that orchestrates pathological osteoresorption involves a variety of mediators and several cell types, both as sources and targets of these secreted factors [28]. Our study revealed specific roles for several chemokines. Namely, CCL2, CCL5, and CXCL10 exhibit a similar magnitude of direct osteoclastogenic action, whereas when tested in vitro, CCL5 has the strongest chemotactic effect on OCPs. However, CCL5 was lower in SF than in plasma of patients with RA in vivo, suggesting its significance for the systemic mobilization of myeloid cells rather than their accumulation in the synovial compartment.

The susceptibility of OCPs to chemotactic signals and the increased production of respective chemokines create optimal conditions for their enhanced homing not only to affected joints but also at the systemic level [29]. Immunophenotyping of PB cells from patients with RA showed a moderate expansion of the total monocyte pool (CD11b^+^CD14^+^), with high osteoclastogenic activity. Furthermore, frequencies of OCP subpopulations expressing chemokine receptors (CCR1, CCR2, CCR4, CXCR3, CXCR4) were significantly increased. These subpopulations, specifically CD3^−^CD19^−^CD56^−^CD11b^+^CD14^+^CXCR4^+^ cells, were positively associated with the indicators of disease severity (i.e., levels of TNF-α, chemokines, autoantibodies, and CTX) and further expanded in the synovial compartment. Moreover, the reduction of the CXCR4^+^ subset in patients treated with DMARDs pointed to the potential application of its frequency as an indicator of a good therapeutic response. Inactivation of CXCR4 has been shown to effectively ameliorate symptoms in murine collagen-induced arthritis, a mouse model of RA [30]. Although CXCL12, an exclusive CXCR4 ligand, has also been implicated in the pathogenesis of several skeletal disorders, including RA [31], we found no difference in CXCL12 levels between plasma and SF of patients with RA, nor did we find a difference compared with the CTRL group. In our in vitro assay, CXCL12 did not show a significant osteoclastogenic or chemotactic effect. Studies in which researchers observed a chemotactic effect of CXCL12 were focused mainly on bone marrow and not on PB OCPs [32, 33]. Thus, in the systemic circulation, involvement of the CXCL12-CXCR4 axis in RA seems to depend on receptor rather than ligand modulation.

In contrast to CXCL12, several tested chemokines (CCL2, CCL4, CXCL10) were elevated in plasma and enriched in SF, indicating that they are produced by cells within the synovial compartment, from whence they spill over into the circulation proportionately to the intensity of the disease [34]. Although aware that a comparison between arthritic and CTRL SF would provide better insights into the changes of the chemokine ligand/receptor profile associated with arthritis, we were unable to obtain appropriate CTRL SF, owing to the limited amount and low cellularity in healthy synovial joints. In addition, arthrocentesis in patients with arthritis was performed for therapeutic evacuation of SF, whereas SF aspiration from healthy subjects has no ethical justification, considering the pain and discomfort involved as well as the risk of articular tissue damage, infection, and joint dysfunction [35]. On the basis of increases in proportions of CCR1^+^ and CCR2^+^ DP subsets, we propose that the CCR1- and CCR2-mediated chemotaxis (by CCL2 and CCL4 ligands) is important for joint-specific recruitment of OCPs. CCR2, expressed in lymphoid and myeloid cells, is important for the recruitment of cells from several lineages, including monocytes, into the joints [27, 36, 37]. In an in vitro study, Lebre et al. suggested that CCR1 blockade might be effective in inhibiting the migration of monocytes toward the synovial compartment in RA [27]. Among DP SFMCs, the CCR4^+^ population was not enriched, indicating that CCR4 is not crucial for accumulation of monocytes in arthritic joints, as opposed to its importance for T lymphocytes [38]. However, circulatory CCR4^+^ OCPs were significantly suppressed during anti-TNF therapy. There are several functional links between TNF-α and chemokine effects that can be modulated by TNF blockade, including TNF-mediated vascular activation that promotes leukocyte adhesion and chemotaxis [39], as well as TNF-induced chemokine secretion by resident and infiltrating inflammatory cells [27]. The dynamic changes of the chemokine receptor profile by anti-TNF treatment observed in our study may reduce OCP susceptibility to chemokine signals and offer a powerful tool to control disease progression.

In addition to providing chemotactic signals, chemokines may act additively to M-CSF/RANKL stimulation to enhance OC differentiation. In our in vitro conditions, we observed an increased number of differentiated bone-resorbing OCs under CCL2, CCL5, and CXCL10 treatment. Studies on isolated human PB monocytes using different culture systems pointed to a direct osteoclastogenic effect of several chemokines, including CCL2 and CCL5, through autocrine and paracrine loops [12, 40, 41]. Besides a direct osteoclastogenic effect, chemokines may be involved in ACPA-mediated OC differentiation [42]. These noncanonical pathways of OC differentiation are specifically important in inflammatory settings, when chemokines as well as proinflammatory cytokines (TNF-α and IL-1) substitute RANKL and M-CSF to augment OC differentiation [43]. A RANKL surplus has not been observed in some animal models of inflammation-induced bone loss, explaining why blocking of the canonical M-CSF/RANKL-mediated osteoclastogenic pathway does not achieve clinical benefit [13]. Negative feedback mechanisms initiated during excessive inflammation may cause refractoriness to M-CSF by ectodomain shedding of CD115 on early human OCPs [25]. This is in line with our observation of significantly reduced CD115 membrane expression in SF-derived monocytes from patients with RA as well as patients with PsA. However, reduced M-CSF expression was not associated with downregulation of RANK expression, suggesting that OC differentiation proceeded despite negative feedback, which was possibly overridden by the excess of other proinflammatory osteoclastogenic signals.

Chemotactic signaling is thought to be important in the pathogenesis of RA, and various chemokines, along with their respective receptors, have been implicated in leukocyte ingress into inflamed synovia [8]. In spite of tremendous efforts to identify immunological abnormalities in RA, a clear association between specific immune cell populations and RA disease activity measures has not been identified, especially for innate immune cells. The majority of studies have dealt with cells involved in adaptive immunity, especially CD4^+^ T lymphocytes [44]. Substantial expression of chemokine receptors on PB monocytes, and more specifically on OCPs, may be particularly important for RA pathogenesis because chemokines initiate several cellular responses crucial for the destructive effects on bone tissue. Although much is known regarding chemokine ligand and receptor profiles in patients with RA, blocking therapy generally did not yield the expected results, thus indicating that more data are required regarding cell- and disease-specific details. The relevant myeloid subpopulations might be more heterogeneous than currently appreciated, with unique markers of OCP subpopulations in various forms of arthritis [13, 28]. By immunophenotyping the chemokine receptor profile on OCPs, and by assessing the in vivo chemokine milieu as well as its in vitro effects, we were able to add to the understanding of the complex inflammatory network present in patients with RA.

## Conclusions

Our results reveal the role of chemokines as potent proinflammatory, chemotactic, and osteoclastogenic factors that may be important for systemic stimulation of OCPs, as well as their increased migration, subsequent tissue homing, and in situ maturation. In addition, we identified arthritis-associated OCP subsets expressing chemokine receptors, which are therefore susceptible to the chemokine gradient, explaining local as well as generalized bone loss seen in RA. Novel insights with regard to the migratory behavior and functional properties of OCPs in response to chemokine signals could open a doorway to new therapeutic targets in RA.

## Additional files

Additional file 1: Figure S1. Increased frequency of osteoclast progenitor cells and subsets expressing chemokine receptors in peripheral blood and synovial fluid samples of patients with RA and PsA. **a** Proportion of OCPs, subsets expressing macrophage colony-stimulating factor receptor (CD115) and RANK in peripheral blood of CTRL subjects and patients with arthritis, and SF samples (SF RA, SF PsA), assessed by flow cytometry. **b** Concentrations of soluble RANKL in SF of arthritic patients, measured by ELISA. **c** Chemokine receptor expression on OCPs in peripheral blood of CTRL subjects and patients with arthritis, and SF of patients with arthritis, assessed by flow cytometry. Values are presented as medians (*middle line*), with *boxes* representing IQR, *whiskers* representing 1.5 times the IQR, and *squares* or *circles* representing outliers. Group-to-group comparisons were performed using a nonparametric Mann-Whitney *U* test, and *p* values <0.05 are shown for comparisons made between PsA and other groups. Previously shown *p* values for comparisons between RA and CTRL groups (Figs. 2 and 3) are not shown again for the sake of visual clarity. (TIF 1768 kb)

Additional file 2: Figure S2. Low level of chemokine gene expression in PBMCs of patients with RA. qPCR analysis of the expression of chemokine genes in PBMCs of CTRL patients and patients with RA, presented as RNA RQ. Values are presented as medians (*middle line*), with *boxes* representing IQR, *whiskers* representing 1.5 times the IQR and *squares* or *circles* representing outliers. Group-to-group comparisons were performed using a nonparametric Mann-Whitney *U* test, *p* values <0.05 are shown. (TIF 987 kb)

Additional file 3: Figure S3. OCs differentiated from PBMCs exhibit OC-specific phenotype and bone-resorbing activity. The number of multinucleated cells expressing TRAP was used for quantification of differentiated OCs under stimulation by M-CSF and RANKL. To further confirm the identity of OCs, parallel cultures were performed and stained for VNR expression. Functional bone-resorbing activity of differentiated OCs was confirmed by pit formation assays using bovine cortical bone slices under the same culture conditions. Presented are representative images of osteoclastogenic cultures of PBMCs from control subjects and patients with RA. In addition, cultures from patients with RA stimulated with CCL5 (10 ng/ml) are shown to confirm the osteoclastogenic effect of CCL5. Red arrows indicate bone resortion pits, formed by active mature osteoclasts. TRAP-stained mature OCs and bovine cortical bone slices were imaged under light microscopy at × 200 magnification. VNR-expressing mature OCs were imaged using a fluorescence microscope at × 200 magnification. (TIF 4477 kb)

## Abbreviations

ACPA: Anticitrullinated protein antibodies
ANOVA: Analysis of variance
APC: Allophycocyanin
CBA: Cytometric bead array
CCL: CC chemokine ligand
CCR: CC chemokine receptor
CDAI: Clinical Disease Activity Index
cDNA: Complementary DNA
cFms: Receptor for colony-stimulating factor-1
CRP: C-reactive protein
CTRL: Control
CTX: Carboxy-terminal telopeptide of type I collagen
CXCL: CXC chemokine ligand
CXCR: CXC chemokine receptor
Cy7: Cyanine 7
DAPI: 4′,6-diamidino-2-phenylindole
DAS28: Disease Activity Score in 28 joints
DMARD: Disease-modifying antirheumatic drug
DP: Double-positive
ELISA: Enzyme-linked immunosorbent assay
ESR: Erythrocyte sedimentation rate
EU: ELISA units
FACS: Fluorescence-activated cell sorting
FITC: Fluorescein isothiocyanate
FSC: Forward scatter
GAPDH: Glyceraldehyde 3-phosphate dehydrogenase
IL: Interleukin
M-CSF: Macrophage colony-stimulating factor
α-MEM: α-Minimal essential medium
N/A: Not applicable
NSAID: Nonsteroidal anti-inflammatory drug
OC: Osteoclast
OCP: Osteoclast progenitor
PB: Peripheral blood
PBMC: Peripheral blood mononuclear cell
PE: Phycoerythrin
PsA: Psoriatic arthritis
qPCR: Quantitative polymerase chain reaction
RA: Rheumatoid arthritis
RANK: Receptor activator of nuclear factor-κB
RANKL: Receptor activator of nuclear factor-κB ligand
RF: Rheumatoid factor
rh: Recombinant human
SF: Synovial fluid
SFMC: Synovial fluid mononuclear cell
SJC: Swollen joint count
SSC: Side scatter
TJC: Tender joint count
TNF-α: Tumor necrosis factor-α
TRAP: Tartrate-resistant acid phosphatase
VAS: Visual analogue scale
VNR: Vitronectin receptor
