# Supplementary figures and images for: Chemokine signals are crucial for enhanced homing and differentiation of circulating osteoclast progenitor cells
Source: Arthritis Res Ther. 2017 Jun 15;19:142. doi: 10.1186/s13075-017-1337-6 (PMC5472975; doi:10.1186/s13075-017-1337-6)

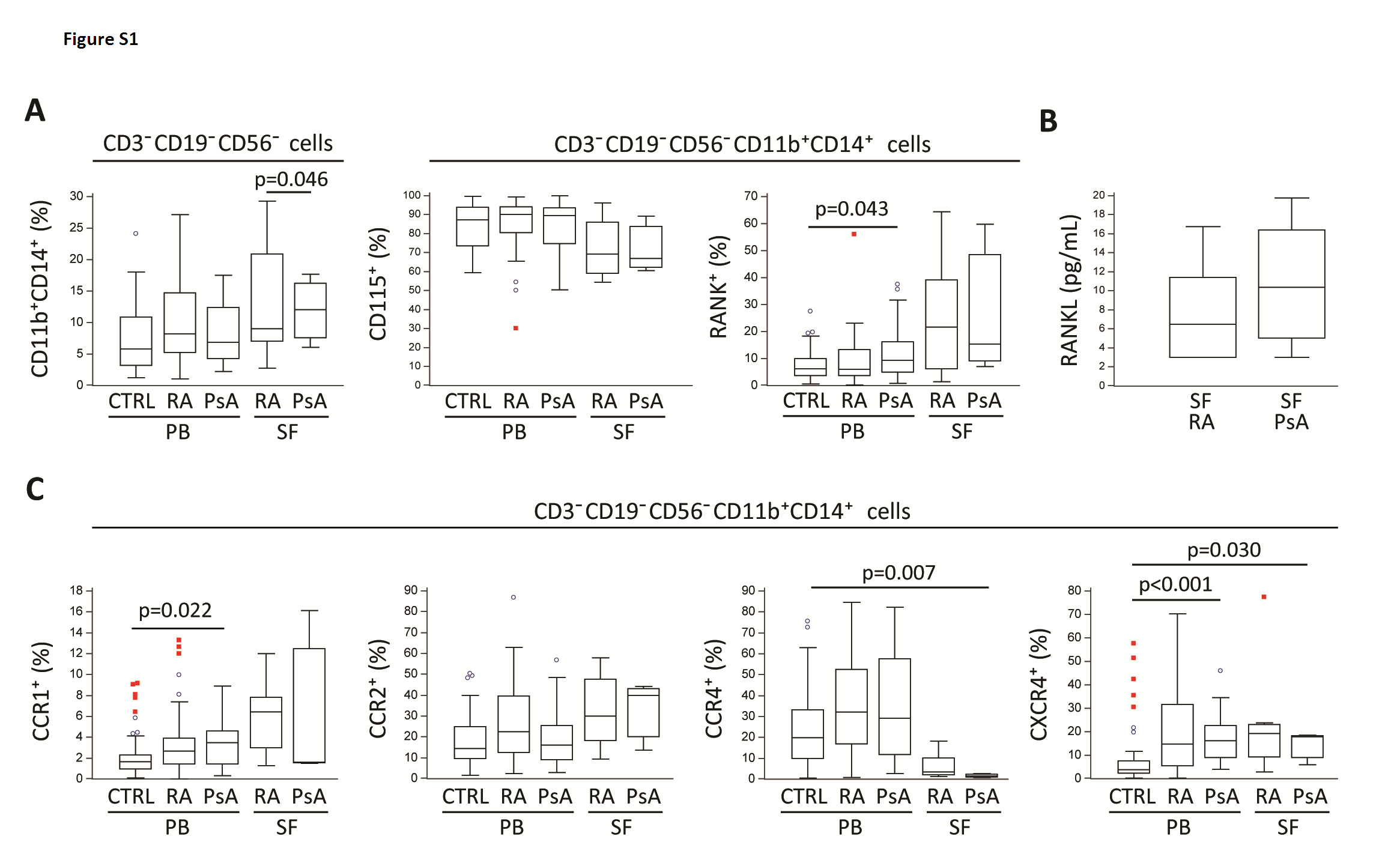

Supplement: Supplementary file 1 — Increased frequency of osteoclast progenitor cells and subsets expressing chemokine receptors in peripheral blood and synovial fluid samples of patients with RA and PsA. a Proportion of OCPs, subsets expressing macrophage colony-stimulating factor receptor (CD115) and RANK in peripheral blood of CTRL subjects and patients with arthritis, and SF samples (SF RA, SF PsA), assessed by flow cytometry. b Concentrations of soluble RANKL in SF of arthritic patients, measured by ELISA. c Chemokine receptor expression on OCPs in peripheral blood of CTRL subjects and patients with arthritis, and SF of patients with arthritis, assessed by flow cytometry. Values are presented as medians (middle line), with boxes representing IQR, whiskers representing 1.5 times the IQR, and squares or circles representing outliers. Group-to-group comparisons were performed using a nonparametric Mann-Whitney U test, and p values <0.05 are shown for comparisons made between PsA and other groups. Previously shown p values for comparisons between RA and CTRL groups (Figs. 2 and 3) are not shown again for the sake of visual clarity. (TIF 1768 kb) [file 13075_2017_1337_MOESM1_ESM.tif]

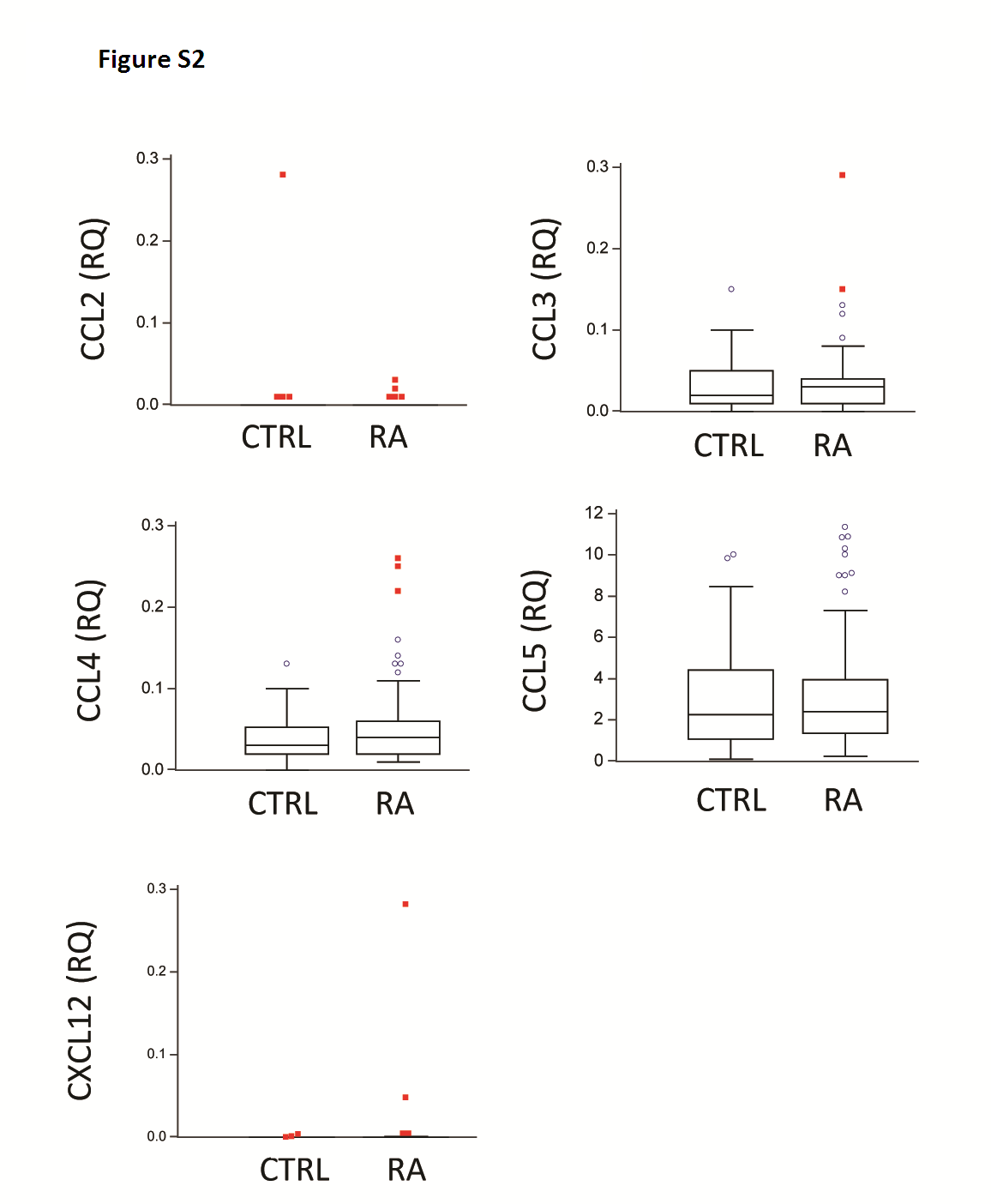

Supplement: Supplementary file 2 — Low level of chemokine gene expression in PBMCs of patients with RA. qPCR analysis of the expression of chemokine genes in PBMCs of CTRL patients and patients with RA, presented as RNA RQ. Values are presented as medians (middle line), with boxes representing IQR, whiskers representing 1.5 times the IQR and squares or circles representing outliers. Group-to-group comparisons were performed using a nonparametric Mann-Whitney U test, p values <0.05 are shown. (TIF 987 kb) [file 13075_2017_1337_MOESM2_ESM.tif]

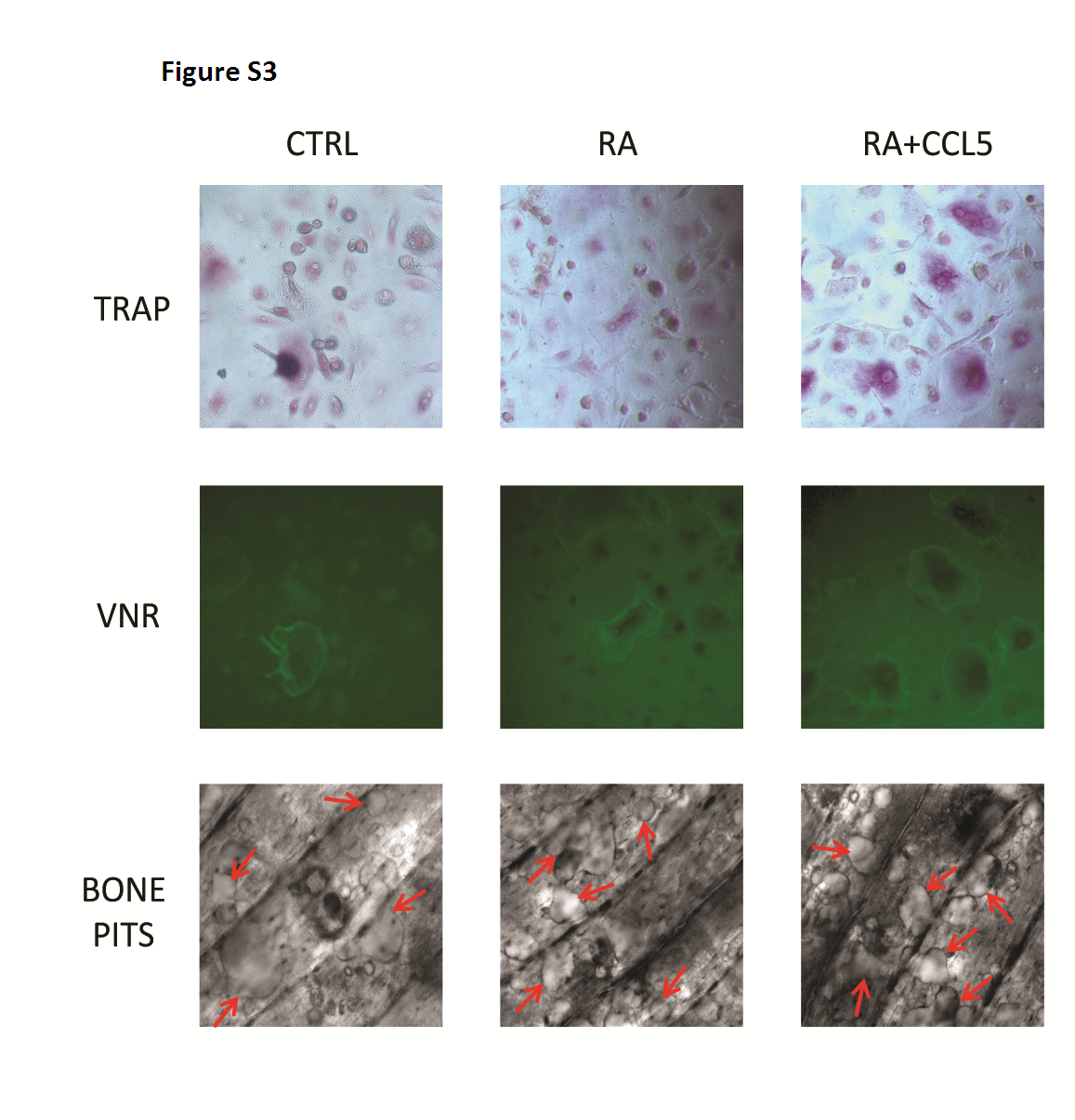

Supplement: Supplementary file 3 — OCs differentiated from PBMCs exhibit OC-specific phenotype and bone-resorbing activity. The number of multinucleated cells expressing TRAP was used for quantification of differentiated OCs under stimulation by M-CSF and RANKL. To further confirm the identity of OCs, parallel cultures were performed and stained for VNR expression. Functional bone-resorbing activity of differentiated OCs was confirmed by pit formation assays using bovine cortical bone slices under the same culture conditions. Presented are representative images of osteoclastogenic cultures of PBMCs from control subjects and patients with RA. In addition, cultures from patients with RA stimulated with CCL5 (10 ng/ml) are shown to confirm the osteoclastogenic effect of CCL5. Red arrows indicate bone resortion pits, formed by active mature osteoclasts. TRAP-stained mature OCs and bovine cortical bone slices were imaged under light microscopy at × 200 magnification. VNR-expressing mature OCs were imaged using a fluorescence microscope at × 200 magnification. (TIF 4477 kb) [file 13075_2017_1337_MOESM3_ESM.tif]
